# Correlation of bevacizumab-induced hypertension and outcome in the BOXER study, a phase II study of capecitabine, oxaliplatin (CAPOX) plus bevacizumab as peri-operative treatment in 45 patients with poor-risk colorectal liver-only metastases unsuitable for upfront resection

**DOI:** 10.1038/bjc.2012.152

**Published:** 2012-04-24

**Authors:** A Dewdney, D Cunningham, Y Barbachano, I Chau

**Affiliations:** 1 Department of Medicine, Royal Marsden Hospital, Downs Road, Sutton, Surrey SM2 5PT, UK

**Keywords:** hypertension, colorectal, metastatic, bevacizumab, biomarker

## Abstract

**Background:**

Bevacizumab is commonly used in combination with chemotherapy in the treatment of metastatic colorectal cancer, but to date, despite extensive research, no predictive or prognostic biomarkers for bevacizumab have been identified. The development of bevacizumab-induced arterial hypertension has recently been suggested as a potential predictive biomarker.

**Methods:**

Blood pressure was recorded during the BOXER study, a phase II study of capecitabine, oxaliplatin (CAPOX) plus bevacizumab as peri-operative treatment in 45 patients with poor-risk colorectal liver-only metastases unsuitable for upfront resection. In this analysis, the development of bevacizumab-induced hypertension was correlated with clinical outcomes.

**Results:**

Fifteen percent of patients developed ⩾grade 1 hypertension while receiving neoadjuvant chemotherapy, and 4% developed grade 3 hypertension. There was no correlation between the development of hypertension and radiological response rate (*P*=0.642), progression-free survival (*P*=0.644) or overall survival (*P*=0.480) in those who developed hypertension compared with those who did not.

**Conclusion:**

Bevacizumab-induced hypertension did not predict radiological response or survival in our study. The results highlight a number of important issues regarding the use of hypertension as a biomarker.

Angiogenesis is the process of new blood vessel formation and is critical for tumour cell survival, growth, invasion and metastases. To date, bevacizumab, a humanised monoclonal antibody to VEGF-A, has been one of the most successful anti-angiogenic agents in the treatment of metastatic colorectal cancer (mCRC). The addition of bevacizumab to combination chemotherapy has demonstrated prolonged progression-free survival (PFS) and overall survival (OS) in the first- and second-line setting (Hurwitz *et al*, 2004; Kabbinavar *et al*, 2005; Saltz *et al*, 2008). With the introduction of targeted agents, the relevance of prognostic and predictive markers in patient selection has become increasingly relevant. However, despite extensive research, the mechanisms responsible for the antitumour activity of antiangiogenic agents is not fully understood, and although many potential biomarkers have been proposed and investigated none have yet been validated for clinical use.

Hypertension is one of the most common side effects of bevacizumab; in the pivotal Hurwitz study, all-grade hypertension was demonstrated in 22.4% of those treated with irinotecan, fluorouracil and leucovorin (IFL) plus bevacizumab compared with 8.3% who received IFL alone (Hurwitz *et al*, 2004). This has been consistently demonstrated in phase II/III clinical trials of bevacizumab in the treatment of colorectal cancer (Table 1). A recent meta-analysis demonstrated that the relative risk of colorectal cancer patients developing grade 3/4 hypertension when treated with bevacizumab was 4.87, *P*=0.0001 (Loupakis *et al*, 2010).

A number of retrospective studies have suggested that the development of bevacizumab-induced arterial hypertension may correlate with clinical outcome in colorectal cancer patients (Table 2). These results are in contrast to a meta-analysis of six large randomised controlled trials of bevacizumab in combination with chemotherapy across a number of tumour types. Of the two colorectal studies included in the meta-analysis, only the AVF2107g trial of IFL, with or without bevacizumab, demonstrated that the development of hypertension predicted for PFS and OS (Hurwitz *et al*, 2004).

We previously conducted a multicentre phase II study of capecitabine, oxaliplatin (CAPOX) plus bevacizumab as peri-operative treatment in 45 patients with poor-risk colorectal liver-only metastases unsuitable for upfront resection (BOXER study) (Wong *et al*, 2011). The overall response rate was 78% (95% CI: 63–89%), which allowed 12/30 (40%) patients with initial non-synchronous unresectable CLM to be converted to resectable. In addition, 10/15 (67%) patients with synchronous resectable CLM underwent liver resection, with four patients being observed to have excellent response to treatment. In light of the emerging data suggesting hypertension as a potential biomarker, we analysed the patients treated within the BOXER trial to assess whether the development of hypertension was associated with outcome in this prospective multicentre phase II study.

## Materials and methods

To be eligible for the study, patients had to have a recorded blood pressure of <145/90 mm Hg^−1^ before commencing treatment and no evidence of proteinuria at baseline as defined by >1 *g* of protein per 24 h by a 24-hour urine collection. Blood pressure measurements were taken after the patient had been in a resting position for ∼5 min and performed before each cycle; repeated measurement of blood pressure for verification was undertaken if the initial reading was 140 mm Hg systolic and/or 90 mm Hg diastolic. Worse-grade toxicity was assessed using common toxicity criteria adverse event (NCI-CTCAE) v3.0, and malignant hypertension was classified as a targeted adverse event. Patients received oxaliplatin (130 mg m^−2^) and bevacizumab (7.5 mg kg^−1^) intravenously on day 1 every 3 weeks. Capecitabine was given orally at a dose of 1700, mg m^−2^ per day in two split doses for 14 days repeated every 3 weeks. No dose reductions of bevacizumab were made, and toxicity attributable to bevacizumab required bevacizumab treatment to be withheld. Any missed doses of bevacizumab were not made up. Treatment response and operability was reassessed after every 12 weeks of treatment, and those whose liver metastases became operable proceeded to metastatectomy. After recovery from surgery, patients received another 12 weeks of CAPOX plus bevacizumab at the same dose schedule as preoperative block.

Hypertension was prospectively recorded using CTCAE v3.0; for the purpose of analysis hypertension was defined as grade ⩾1 hypertension (asymptomatic, transient (<24 h) increase by 20 mm Hg or to >150/100 if previously normal: intervention not indicated). Fisher’s exact test was used to analyse the association between the development of hypertension and radiological response. The Kaplan–Meier method was used to estimate OS and PFS, and groups were compared using the log-rank test; separate analyses were performed for each grade of hypertension (grade 1–3).

## Results

The median number of bevacizumab-containing cycles was eight. For those patients proceeding to liver resection, the median number of preoperative cycles of CAPOX plus bevacizumab was four. Overall, the treatment was well tolerated and grade 3/4 toxicity was low. Fifteen percent of patients developed ⩾grade 1 hypertension while receiving neoadjuvant chemotherapy. Only 4% developed grade 3 hypertension. In four cases, the hypertension was recorded on a single occasion, and in the remaining cases it occurred more than once (range 1–6). The onset of hypertension was early (cycle 1–3) in 75% of cases. Three patients required antihypertensive therapy during their chemotherapy. Of those who developed hypertension, three had a past medical history of controlled hypertension and were on at least one antihypertensive at trial entry (range 1–3).

There was no difference in radiological response between those who developed hypertension and those who did not (71% *vs* 78% *P*=0.642). No statistical comparison was possible between the grade of hypertension and outcome or individual responses (CR, PR, SD, PD) because of the small numbers.

**Figure 1:**
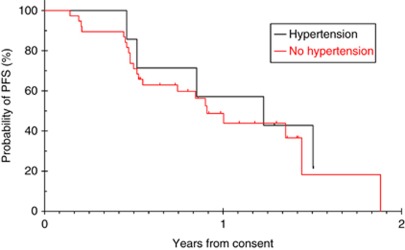
Kaplan–Meier analysis of progression-free survival.

Progression-free survival at 12 months was 49% in the non-hypertension group *vs* 57% in the hypertension group (*P*=0.664) (Figure 1). In addition, there was no difference in PFS according to the grade of hypertension (*P*=0.886).

**Figure 2:**
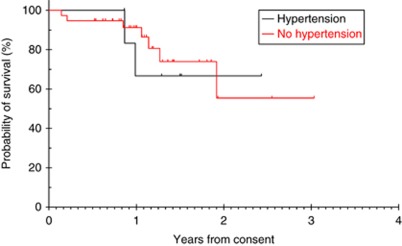
Kaplan–Meier estimate of overall survival.

Overall survival at 12 months was 67% in the hypertension group and 91% in the non-hypertensive group (*P*=0.826) (Figure 2). There was no difference in OS according to grade of hypertension (*P*=0.480).

## Discussion

Our results demonstrate that hypertension is a common side effect of bevacizumab, occurring in 15% of patients; however, in contrast to recent data, the development of hypertension was not predictive of outcome in this study. Our study is limited by the small patient numbers, and our conflicting results may be related to the high overall response rate or simply the low incidence of hypertension in this study. The overall rate of hypertension in this study was lower than the previously published rates, potentially a consequence of the highly selected patient population. Our findings highlight the complexities of the use of hypertension as a potential biomarker, in particular the presence of coexistent hypertension, complicating interpretation of retrospective analyses.

The data suggesting an association between the development of hypertension and outcome have been mostly, similar to ours, retrospective analyses of blood pressure, with considerable variation in the method of recording and definition of hypertension. It is well documented that blood pressure is affected by multiple factors, which can be patient or operator related, and even small changes in blood pressure cuff position or bladder volume at the time of recording can affect readings. The CTCAE grading for hypertension is also relatively subjective, which may contribute to the lack of reproducibility demonstrated in these studies. Alternatively, more accurate methods of blood pressure monitoring include ambulatory blood pressure measurement; however, the feasibility of ambulatory monitoring is limited in daily clinical practice.

It remains unclear whether baseline blood pressure, the rate of rise, the absolute rise, the timing of onset or the duration of hypertension are all equally relevant factors. In particular, the clinically important cutoff values for percentage rise in blood pressure have yet to be established. In one study, a predictive effect was demonstrated in patients with grade ⩾2–4 hypertension (De Stefano *et al*, 2011), and in another the benefit was seen with any grade of hypertension (Osterlund *et al*, 2011). Certainly, the most common rise in blood pressure does not appear to be very high (10–20 mm Hg systolic), and the incidence of hypertensive crisis in most studies is low (0.1%). In addition, the median time to onset of hypertension is not well documented.

Before the emergence of evidence demonstrating *KRAS* mutation as a predictive biomarker for the lack of response to anti-EGFR antibodies, the severity of skin rash developed by patients was the only consistent biomarker for response. The EVEREST study demonstrated that dose escalation of cetuximab in patients who did not initially develop an intense rash resulted in a higher incidence of grade 3 skin reactions, which correlated with an increase in response rates (Van Cutsem *et al*, 2007). On a similar basis, the prevalence of hypertension with anti-angiogenics led to the suggestion that dose titration of bevacizumab until blood pressure elevation may lead to better anti-tumour efficacy and improved outcomes (Maitland and Ratain, 2006). Although the incidence of hypertension does appear to increase with higher doses of bevacizumab (Kabbinavar *et al*, 2003), there is currently no evidence to suggest that this results in improved outcomes.

Patients receiving anti-angiogenic agents should have their blood pressure monitored throughout the treatment, with more frequent assessments during the first cycle of treatment. Trial guidelines and bevacizumab prescribing information routinely recommend treating bevacizumab-induced hypertension with an ACE inhibitor or calcium channel blocker and continuing treatment rather than reducing the dose of the bevacizumab, so as not to deny patients potential benefit. Little is known about the effect of treating bevcizumab-induced hypertension and whether this could potentially negate the benefit of bevacizumab.

Certainly, we remain in need of a biomarker for bevacizumab; nevertheless, despite recent data we are left with a number of unanswered questions regarding the role of hypertension as a biomarker. In particular, how do we, and more importantly should we, prospectively validate an adverse event as a biomarker? The clinical impact of severe hypertension is not insignificant, and is associated with the risk of cardiovascular complications and arterial thromboembolic events (Ranpura *et al*, 2010, 2011). With a median survival of patients with mCRC up to 24 months, it is becoming increasingly relevant to focus on potential long-term side effects in this group of patients.

Factors that predict bevacizumab-induced hypertension could assist in the prospective selection of patients for treatment; in a breast cancer study, VEGF genotype predicted clinically significant hypertension and those with VEGF-1498TT and VEGF-634 CC were largely protected from serious hypertension (Schneider *et al*, 2008). However, these studies have yet to be replicated in colorectal cancer.

There is no doubt that the identification of hypertension as a potential biomarker has increased our understanding of the mechanism of action of anti-angiogenic agents, yet how we implement this knowledge remains uncertain. A number of prospective studies currently evaluating factors such as flow-mediated vasodilation, nitric oxide activity and vascular dysfunction are ongoing; however, given the complexities associated with the use of an adverse event as a biomarker, significant research is still needed to identify alternative biomarkers for bevacizumab.

## Figures and Tables

**Table 1:** Bevacizumab associated hypertension in colorectal studies

**Study**	**Drug**	**Dose bevacizumab**	**Number of patients**	**Rate all-grade hypertension (%)**	**Rate grade3/4 hypertension (%)**
*Adjuvant*					
NSABP C-08 Allegra *et al* (Allegra *et al*, 2009)	mFOLFOX mFOLFOX/bevacizumab	5 mg kg^−1^	1321 1326	NR NR	1.8 12
					
*1st line*					
Hurwitz *et al* (Hurwitz *et al*, 2004)	IFL/bevacizumab IFL/placebo	5 mg kg^−1^	402 411	22.4 8.3	11 2
N016966 Saltz *et al* (Saltz *et al*, 2008)	FOLFOX/XELOX FOLFOX/XELOX+bevacizumab	7.5 mg kg^−1^ (XELOX) 5 mg kg^−1^ (FOLFOX)	701 699	NR NR	1 4
Kabbinavar *et al* (Kabbinavar *et al*, 2005)	FU/LV/placebo FU/LV/bevacizumab	5 mg kg^−1^	105 104	5 32	16 3
BRITE Kozloff *et al* (Kozloff *et al*, 2009)	Chemotherapy+bevacziumab	5 mg kg^−1^	1953	22^a^	NR
					
*2nd line*					
E3200 (Giantonio *et al*, 2007)	FOLFOX FOLFOX/bevacizumab Bevacizumab	10 mg kg^−1^ 10 mg kg^−1^	291 289 243	NR NR NR	1.8 6.2 7.3

Abbreviations: IFL=irinotecan, fluorouracil and leucovorin; NR=not reported.

a HTN requiring medication.

**Table 2:** Bevacizumab therapy induced hypertension and outcome in colorectal cancer

					**ORR (%)**			**PFS (months)**			**OS (months)**		
**Author**	**Treatment**	**No. of patients**	**Dose**	**Rate (%)** **Hypertension**	**Hypertensive group**	**Non hypertensive group**	***P*** **-value**	**Hypertensive group**	**Non hypertensive group**	***P*** **-value**	**Hypertensive group**	**Non hypertensive group**	***P*** **-value**
Osterlund *et al*, 2011	Chemo+bevacizumab	101	5 mg kg^−1^ 2 weekly or 7.5 mg kg^−1^ 3 weekly	56^a^	30	20	0.025	10.5	5.3	0.008	25.8	11.7	<0.001
Scartozzi *et al*, 2009	FOLFIRI+bevacizumab	39	5 mg kg^−1^ 2 weekly	20^b^	75 (6/8)	32 (10/31)	0.04	14.5	3.1	0.04	Not reached	15.1	—
De Stefano *et al*, 2011	Chemo+bevacizumab	74	NR	17.6^c^	84.6	42.6	0.006	15.1	8.3	0.04	35.5	26.7	0.2

Abbreviations: NR=not reported; ORR=overall response rate; PFS=progression-free survival.

a ⩾Grade 1.

b Grade 2–3.

c Grade 2–4.
